# Prevalence and risk factors of sarcopenic obesity in Thai people with diabetes: A cross-sectional study

**DOI:** 10.1016/j.obpill.2026.100249

**Published:** 2026-02-07

**Authors:** Thanapat Limpaarayakul, Kasidid Lawongsa, Methavee Poochanasri, Nattapol Sathavarodom, Apussanee Boonyavarakul, Parinya Samakkarnthai

**Affiliations:** a Division of Cardiology, Department of Medicine, Phramongkutklao Hospital, Bangkok, Thailand; b Department of Family Medicine and Outpatient Department, Phramongkutklao Hospital, Bangkok, Thailand; c Department of Medicine, Bhumibol Adulyadej Hospital, Bangkok, Thailand; d Division of Endocrinology, Department of Medicine, Phramongkutklao Hospital, Bangkok, Thailand

**Keywords:** Sarcopenic obesity, Type 2 diabetes mellitus, Prevalence, Risk factors

## Abstract

**Background:**

Sarcopenic obesity is an age-related condition characterized by a simultaneous decline in muscle mass and function and by increased adipose tissue. Its prevalence, particularly in Asian populations with type 2 diabetes mellitus (T2DM), remains unclear. This study aimed to determine the prevalence and risk factors associated with sarcopenic obesity in Thai people with T2DM.

**Methods:**

This cross-sectional, observational study included 329 Thai adults with established type 2 diabetes mellitus (T2DM), consecutively recruited from an outpatient clinic. Participants underwent standardized assessments, including bioelectrical impedance analysis, physical performance testing, and laboratory evaluations. Obesity was defined based on percentage body fat derived from bioelectrical impedance analysis, rather than body mass index. The study aimed to determine the prevalence of sarcopenic obesity and to identify associated risk factors. Univariate and multivariate logistic regression analyses were performed to identify independent predictors after adjustment for potential confounders.

**Results:**

The prevalence of sarcopenic obesity in T2DM was 16.4%, with rates of 15.4% in males and 17.5% in females. Sarcopenia was present in 23.7% of participants, including 7.3% with sarcopenia alone (without obesity). Obesity without sarcopenia was reported in 61.1% (95% CI: 55.7, 66.2). Factors significantly associated with a higher risk of sarcopenic obesity included older age (adjusted OR 1.08, 95% CI 1.01–1.15, P < 0.001) and smaller calf circumference (aOR 1.35, 95% CI 1.11–1.67, P < 0.001). Protective factor included greater handgrip strength (aOR 0.93, 95% CI 0.87–0.99, P < 0.001).

**Conclusions:**

The study found a high prevalence of sarcopenic obesity among Thai people with T2DM. Older age and smaller calf circumference were identified as key risk factors. Higher muscle strength was associated with a lower risk of developing sarcopenic obesity.

## Graphical abstract

**Figure:**
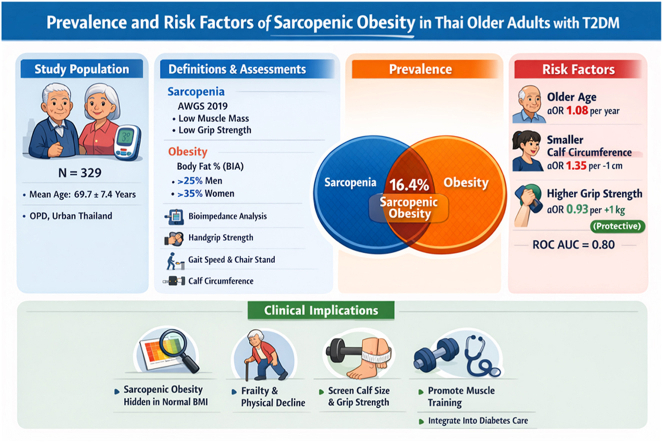

## 1 Introduction

Sarcopenic obesity is characterized by a concurrent decline in muscle mass and function alongside an increase in adipose tissue. This condition is becoming a growing concern among older adults due to its significant health consequences, including impacts on mortality, comorbidities, and the risk of developing geriatric syndromes [1]. The definition of sarcopenic obesity refers to the coexistence of obesity and sarcopenia [2]. The definition of sarcopenia differs between two organizations: the Asian Working Group for Sarcopenia (AWGS) and the European Working Group on Sarcopenia in Older People (EWGOS), which use different screening criteria and cut-points due to the anthropometric characteristics of the Asian population. However, both organizations share a similar testing process that requires patients to undergo assessments of grip strength, walking speed, 5-time chair stand test, and skeletal muscle mass, which is measured using bone mass density machines (dual-energy X-ray absorptiometry or DXA), magnetic resonance imaging (MRI), computed tomography (CT scan), or bioelectrical impedance analysis (BIA) for body composition measurement. The prevalence of sarcopenic obesity varies according to different definitions, estimated at around 10–27% using EWGOS [3].

Obesity can also cause sarcopenia independently through adipose tissue-dependent metabolic derangements, such as oxidative stress, inflammation, and insulin resistance, which negatively affect muscle mass [4]. The definition of obesity varies across studies, with some using BMI and others percent body fat, each with distinct cutoff points for different sexes and ethnicities. As a result, the prevalence of sarcopenic obesity is not well established. Sarcopenic obesity also reduces the efficacy of daily life tasks, reduces quality of life, and is associated with higher fasting blood glucose, insulin resistance, blood pressure, plasma lipid abnormalities, a higher mortality rate, cardiovascular disease, and malignancy compared to those who have obesity without sarcopenia [5,6].

Type 2 diabetes mellitus (T2DM) is a complex metabolic condition in which insulin resistance, often associated with obesity and metabolic syndrome, is accompanied by progressive β-cell dysfunction, resulting in hyperglycemia. Continuous hyperglycemia can induce target organ damage (TOD) by increasing the risk of macro- and microvascular disease [7]. Diabetic vasculopathy and the accumulation of advanced glycation end-products (AGEs) may also impair muscle mass and function, leading to sarcopenia [8]. T2DM represents a critical health burden in the elderly population, affecting approximately 25% of people over the age of 65 years; this proportion is expected to further increase in the following decades [9]. In people with T2DM, the risk of sarcopenia is 1.55 times greater than in the general population [10]. Also, People with T2DM with sarcopenic obesity have an increased risk of all-cause mortality [10].

The prevalence of OLLMM (obesity with low lean muscle mass) in the USA was 15.9% of the population. This prevalence was higher among older individuals (8.1% in those aged 20–59 years compared with 28.3% in those aged ≥60 years), with the highest rate (66.6%) in Mexican-American females aged ≥60 years and the lowest rate (2.6%) in non-Hispanic Black males aged 20–59 years. There was a higher prevalence of OLLMM in adults with prediabetes (19.7%) and type 2 diabetes mellitus (T2DM) (34.5%) [11]. In a systematic review and meta-analysis, the prevalence of sarcopenic obesity among Asians was found to be 26% [12]. However, criteria for both sarcopenia and obesity differ. Some studies found that it is significantly associated with decreased glomerular filtration rate, massive proteinuria, and insulin resistance in patients with diabetes [12]. Risk factors such as aging, physical inactivity and a sedentary lifestyle, chronic stress, and consistently high cortisol levels may increase the risk of sarcopenia [1]. However, the prevalence and risk factors of sarcopenic obesity in people with T2DM have not been well studied in Thailand. This cross-sectional study aims to evaluate the prevalence and risk factors of sarcopenic obesity and to assess and prevent complications arising from it in urban areas of Thailand.

## 2 Methods

Between December 2023 and November 2024, a cross-sectional study of 329 patients was conducted at the outpatient department (OPD) clinic of Phramongkutklao Hospital in Bangkok, Thailand. The study included patients with T2DM who were at least 60 years old and willing to participate. Individuals with a history of active cancer, uncontrolled underlying diseases, current use of medications affecting muscle mass (e.g., corticosteroids), limited mobility due to neurological impairments, or inability to undergo physical tests were excluded. The study received approval from the Royal Thai Army Institutional Review Board under reference R080h/66, and written informed consent was obtained from all participants before their participation.

### 2.1 Sarcopenia

The Asian Working Group for Sarcopenia (AWGS) defines sarcopenia as a loss of muscle mass, accompanied by decreased muscle strength and/or physical performance. The AWGS 2019 screening and diagnostic criteria are as follows [13]^.^

#### 2.1.1 Screening

Calf circumference (CC): measured with a tape measure in a seated position with feet on the floor and the non-dominant leg at 90°. Men with a maximum CC of <34 cm and women with a maximum CC of <33 cm are considered to have low CC. SARC-CalF consists of a five-question SARC-F questionnaire and an additional calf circumference measurement. Men with a maximum CC of <34 cm and women with a maximum CC of <33 cm are assigned an additional 10 points. Total scores range from 0 to 20. A score of 11 or higher indicates a positive sarcopenia screening.

#### 2.1.2 Diagnosis

Muscle Strength Measurement: A digital hand dynamometer was used to assess handgrip strength. Participants initially used their dominant hand while seated with a 90-degree elbow flexion and performed three grip tests. The best performance from these tests was recorded in kilograms. According to the AWGS 2019 guidelines, low handgrip muscle strength is < 28.0 kg for men and <18.0 kg for women.

#### 2.1.3 Physical performance measurement

6-m walk test: The gait speed test measures the time required for participants to walk 6 m at a standard pace. Two trials were conducted, and the average was calculated in meters per second. The cut-off for low physical performance is a gait speed of less than 1.0 m/s during the 6-m walk.

5-time chair stand test: ≥12 s. The chair stand time is the number of seconds needed to complete five repetitions of standing up and sitting in a chair. A completion time equal to or greater than 12 s is considered the cutoff for low physical performance.

#### 2.1.4 Muscle mass measurement (Appendicular skeletal muscle mass)

The results were assessed using bioelectrical impedance analysis (InBody 970), with muscle mass calculated as limb muscle mass divided by height squared. AWGS 2019 defines it as less than 7.0 kg/m^2^ in males and less than 5.7 kg/m^2^ in females. Appendicular skeletal muscle mass (ASM), defined as the sum of lean mass in the arms and legs, was estimated using BIA.

### 2.2 Obesity

According to the American Association of Clinical Endocrinology (AACE), obesity is defined as a body fat percentage of ≥25% in men and ≥35% in women [14]^.^ It can also be assessed using a body mass index (BMI) of ≥30 kg/m^2^ or a waist circumference of ≥102 cm in men and ≥88 cm in women. In Thailand, the World Health Organization (WHO) defines obesity as a BMI ≥25 kg/m^2^ or a waist circumference ≥90 cm in men and ≥80 cm in women [15]^.^ In this study, we considered a body fat percentage of ≥25% in men and ≥35% in women, as measured by BIA, to be consistent with AACE guidelines.

### 2.3 Waist circumference

The WHO and the International Diabetes Federation recommend measuring waist circumference at the midpoint between the last rib and the iliac crest (pelvic bone).

### 2.4 Diabetes mellitus

According to the American Diabetes Association, diabetes is diagnosed using either two different tests or the same test performed twice. The criteria include: Fasting plasma glucose (FPG) ≥ 126 mg/dL, 2-h plasma glucose (PG) ≥ 200 mg/dL, HbA1c ≥ 6.5%, or symptoms of diabetes plus random plasma glucose ≥200 mg/dL.

### 2.5 Frailty phenotype

According to the frailty phenotype from Fried et al. [16], the criteria are [1] Unintentional weight loss of >10 lbs (≥4.5 kg) or ≥5% of body mass in the last year (obtained from patient, caregiver, or medical records) [2]; Weakness (assessed by handgrip strength; interpretation of results takes into account sex and BMI) [3]; Exhaustion (audited information based on two questions from the Center for Epidemiological Studies Depression (CES-D) scale; a score of 1 (fatigue or exhaustion felt rarely or not at all) to 4 (fatigue or exhaustion felt most of the time); a score of 3 or 4 indicates a positive test for decreased physical activity) [4]; Slow gait (walking time over a distance of 4.5 m; interpretation of results takes into account sex and height) [5]; Low physical activity (weekly energy expenditure rate calculated based on the modified Minnesota Leisure Time Activity Questionnaire).

### 2.6 Statistically analyses

Mean and standard deviation represent continuous variables, whereas observed counts and percentages represent categorical variables. Prevalence rates of sarcopenia and sarcopenic obesity are reported as percentages. When comparing dependent variables across study groups, the independent *t*-test or ANOVA was used as appropriate. The control group comprised participants without sarcopenic obesity, including those with sarcopenia alone, obesity alone, or neither condition. Univariate and multivariate logistic regression analyses were used to examine associations between clinical factors (age, BMI, appendicular skeletal muscle mass [ASM], and sarcopenia) and the prevalence of sarcopenic obesity. Serum hs-CRP levels were summarized as median and interquartile range (IQR) due to a non-normal distribution. Differences across sarcopenic obesity, obesity alone, and sarcopenia alone groups were assessed using the Kruskal–Wallis test. When appropriate, post hoc pairwise comparisons were performed using Dunn’s test with Bonferroni adjustment. All statistical analyses were conducted using SPSS, version 17 for Windows Evaluation Software (SPSS Inc., Chicago, IL). Statistical significance was considered at a *p*-value of less than 0.05.

## 3 Results

**Fig. 1:**
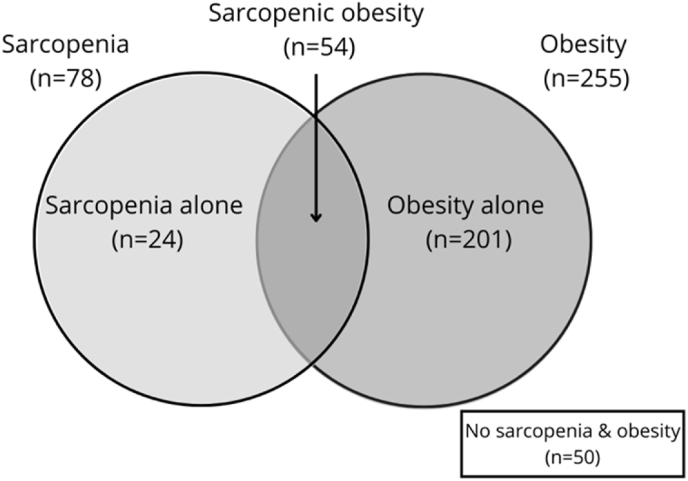
Venn diagram of sarcopenic obesity in people with type 2 diabetes mellitus.

A total of 329 participants with T2DM were included in the study, with 55.0% women and 45.0% men. The participants had a mean age of 69.65 ± 7.39 years and a body mass index (BMI) of 26.09 ± 4.58 kg/m^2^. Using the AWGS 2019 criteria for sarcopenia and obesity, defined by percent body fat, the prevalence of sarcopenic obesity was 16.4% (95% CI: 12.8, 20.8), with 15.4% (95% CI: 10.7, 21.7) in males and 17.5% (95% CI: 12.3, 24.2) in females. The prevalence of sarcopenia alone was 7.3% (95% CI: 4.9, 10.7), obesity without sarcopenia was 61.1% (95% CI: 55.7, 66.2), and the control group constituted 15.2%. The prevalence of sarcopenia, including both obesity and non-obesity, was 23.7% (see Fig. 1). Patient characteristics are detailed in Table 1. The mean age of the sarcopenic obesity group was 74.81 ± 8.37 years, significantly higher than that of the obesity and control groups (P < 0.001). The BMI in the sarcopenic obesity group was 22.96 ± 2.35 kg/m^2^, significantly higher than that of the sarcopenia alone group but notably lower than that of the obesity group. Dyslipidemia was most common in the sarcopenia alone group (26.9%) and the obesity group (90.6%), which were significantly higher than in the other groups (P = 0.010). There were no significant differences among groups regarding diabetes duration, family history, smoking habits, or alcohol consumption (P > 0.05).

**Table 1:** Baseline characteristics of People with T2DM classified by sarcopenia and/or obesity.

Variables	Sarcopenic obesity	Sarcopenia alone	Obesity	Control	P-value
n (%) [95%CI]	54 (16.4%) [12.8%, 20.8%]	24 (7.3%) [4.9%, 10.7%]	201 (61.1%) [55.7%, 66.2%]	50 (15.2%) [11.7%, 19.5%]	
Age (years), mean ± SD	74.81 ± 8.37^a,b^	71.96 ± 7.65	68.21 ± 6.69^a^	68.74 ± 6.21^b^	<0.001∗
Sex, n(%)					
Male	26 (48.2)	7 (29.2)	109 (54.2)	27 (54.0)	0.124
Female	28 (51.8)	17 (70.8)	92 (45.8)	23 (46.0)	
Education, n(%)					
No education/Primary school	18 (33.3)	7 (29.2)	42 (21.0)	8 (16.0)	0.028∗
Secondary school/diploma	7 (13.0)	7 (29.2)	66 (33.0)	22 (44.0)	
Bachelor degree or higher	29 (53.7)	10 (41.7)	92 (46.0)	20 (40.0)	
Body mass index (kg/m^2^), mean ± SD	22.96 ± 2.35^a,b^	19.87 ± 2.88^a,c,d^	28.40 ± 3.95^b,c,e^	23.15 ± 2.57^d,e^	<0.001∗
BMI classification, n(%)					
<18.5	2 (3.7)	5 (20.8)	0 (0.0)	2 (4.0)	<0.001∗
18.5–22.9	24 (44.4)	16 (66.7)	8 (4.0)	21 (42.0)	
23.0–24.9	21 (38.9)	1 (4.2)	31 (15.4)	15 (30.0)	
≥25.0	7 (13.0)	2 (8.3)	162 (80.6)	12 (24.0)	
Underlying disease, n(%)					
Hypertension	41 (75.9)	17 (70.8)	166 (82.6)	36 (72.0)	0.230
Dyslipidemia	47 (87.0)	22 (91.7)	182 (90.6)	37 (74.0)	0.015∗
Myocardial infarction/Coronary artery disease	8 (15.4)	3 (13.0)	16 (8.4)	5 (10.9)	0.488
Duration of DM (years), median (IQR)	10 (5, 20)	10 (6.5, 20)	10 (5, 20)	11 (5, 24)	0.303
Family history of DM, n(%)	36 (66.7)	15 (62.5)	136 (67.7)	38 (76.0)	0.605
Smoking, n(%)	2 (3.7)	0 (0.0)	10 (4.9)	4 (8.0)	0.484
Frequency of alcohol consumption, n(%)					
None	51 (94.4)	22 (91.7)	161 (80.1)	38 (76.0)	0.086
Rarely	3 (5.6)	2 (8.3)	36 (17.9)	12 (24.0)	
Everyday	0 (0.0)	0 (0.0)	4 (2.0)	0 (0.0)	0.086
Herbal use, n(%)	5 (9.3)	0 (0.0)	18 (9.0)	4 (8.0)	0.498
Weight loss in 1 year, n(%)	32 (59.3)	14 (58.3)	107 (53.2)	33 (66.0)	0.406

a,b,c: Identical letters indicate statistically significant differences between groups at the 0.05 level.

∗Statistically significant at the 0.05 level (α = 0.05).

The physical performance test revealed that the sarcopenic obesity and sarcopenia alone groups had median SARC-F scores significantly higher than those of the other groups (P = 0.010), with 22.2% and 33.3% of participants scoring four or higher, respectively. Frailty was most common in the sarcopenic obesity group (31.5%), followed by the sarcopenia alone group (29.2%), the control group (14.0%), and the obesity group (10.5%). These differences were statistically significant (P < 0.001). However, there were no statistically significant differences in exercise frequency, walking, or fatigue (P > 0.05; Table 2). Body composition analysis indicated that the sarcopenic obesity group had lower means for weight (56.38 ± 7.36 kg), height (156.66 ± 8.54 cm), elevated diastolic BP (65.06 ± 11.18 mmHg), calf circumference (33.64 ± 2.18 cm), neck circumference (36.51 ± 2.91 cm), hand grip strength (18.42 ± 7.39 kg), and skeletal muscle mass index (5.72 ± 0.74 kg/m^2^) compared with the obesity and control groups (P < 0.05). Conversely, the sarcopenic obesity group had significantly longer times for the 4.5-m walk (5.97 s [IQR 4.96, 7.39]) and the 5-time chair stand test (18.56 ± 6.19 s) compared with the obesity and/or control groups (P < 0.05). The waist circumference in the sarcopenic obesity group (88.74 ± 7.44 cm) was significantly greater than that of the sarcopenia alone group but notably smaller than that of the obesity group, as detailed in Table 3.

**Table 2:** Physical fitness measurement of People with T2DM classified by sarcopenia and/or obesity.

Variables	Sarcopenic obesity	Sarcopenia alone	Obesity	Control	P-value
n (%) [95%CI]	54 (16.4%) [12.8%, 20.8%]	24 (7.3%) [4.9%, 10.7%]	201 (61.1%) [55.7%, 66.2%]	50 (15.2%) [11.7%, 19.5%]	
Frequency of exercise, n(%)					
None	27 (50.0)	10 (41.7)	95 (47.3)	20 (40.0)	0.827
1–2 day/week	5 (9.3)	1 (4.2)	13 (6.5)	3 (6.0)	
3–4 day/week	7 (12.9)	4 (16.7)	43 (21.4)	11 (22.0)	
≥5 day/week	15 (27.8)	9 (37.5)	50 (24.9)	16 (32.0)	
Walking time (hour/week), mean ± SD	14.04 ± 6.16	15.58 ± 6.70	16.20 ± 6.48	16.68 ± 7.16	0.137
Exhaustion, n(%)	22 (40.7)	10 (41.7)	75 (37.3)	16 (32.0)	0.785
SARC-F score, median (IQR)	2 (1, 3)^a,b^	2.5 (1, 4.5)^c,d^	1 (0, 3)^a,c^	1 (0, 2)^b,d^	0.023∗
≥4 score, n(%)	12 (22.2)	8 (33.3)	29 (14.4)	6 (12.0)	0.058
Frailty phenotype, n(%)					
Non-frail	6 (11.1)	0 (0.0)	45 (22.4)	13 (26.0)	
Pre-frailty	31 (57.4)	17 (70.8)	135 (67.2)	30 (60.0)	<0.001∗
Frailty	17 (31.5)	7 (29.2)	21 (10.5)	7 (14.0)	

a,b,c: Identical letters indicate statistically significant differences between groups at the 0.05 level.

∗Statistically significant at the 0.05 level (α = 0.05).

**Table 3:** Body measurement of People with T2DM classified by sarcopenia and/or obesity.

Variables	Sarcopenic obesity	Sarcopenia alone	Obesity	Control	P-value
n (%) [95%CI]	54 (16.4%) [12.8%, 20.8%]	24 (7.3%) [4.9%, 10.7%]	201 (61.1%) [55.7%, 66.2%]	50 (15.2%) [11.7%, 19.5%]	
Weight (kg), mean ± SD	56.38 ± 7.36^a,b,c^	48.9 ± 7.71^a,d,e^	74.89 ± 12.59^b,d,f^	62.53 ± 10.53^c,e,f^	<0.001∗
Height (cm), mean ± SD	156.66 ± 8.54^a,b^	156.88 ± 7.91^c,d^	162.26 ± 8.78^a,c^	163.95 ± 8.14^b,d^	<0.001∗
Systolic blood pressure (mmHg), mean ± SD	128.37 ± 15.27	135.08 ± 17.05	134.47 ± 14.72	134.74 ± 15.50	0.056
Elevated diastolic BP (mmHg), mean ± SD	65.06 ± 11.18^a^	65.75 ± 10.31	71.63 ± 11.63^a^	69.80 ± 8.42	0.004∗
Calf circumference (cm), mean ± SD	33.64 ± 2.18^a,b,c^	31.46 ± 2.25^a,d,e^	38.79 ± 3.48^b,d,f^	35.95 ± 3.09^c,e,f^	<0.001∗
Waist circumference (cm), mean ± SD	88.74 ± 7.44^a,b^	76.35 ± 12.98^a,c,d^	99.96 ± 11.18^b,c,e^	87.46 ± 8.92^d,e^	<0.001∗
Neck circumference (cm), mean ± SD	36.51 ± 2.91^a^	35.75 ± 8.42^b^	40.09 ± 3.49^a,b,c^	37.89 ± 3.21^c^	<0.001∗
Walk 4.5 m (sec), median (IQR)	5.97 (4.96, 7.39)^a,b^	6.25 (4.52, 7.86)^c^	5.28 (4.36, 6.52)^a^	4.96 (4.56, 5.53)^b,c^	0.007∗
5-Time chair stand (sec), mean ± SD	18.56 ± 6.19^a,b^	17.21 ± 4.82	15.15 ± 5.44^a^	15.44 ± 4.59^b^	0.006∗
≥12 s, n(%)	45 (88.2)	22 (95.7)	138 (70.4)	37 (77.1)	0.006∗
Hand grip strength (kg), mean ± SD	18.42 ± 7.39^a,b^	16.65 ± 5.76^c,d^	23.69 ± 7.39^a,c^	25.05 ± 8.51^b,d^	<0.001∗
M < 28 kg; F < 18 kg, n(%)	42 (77.8)	21 (87.5)	93 (46.3)	18 (36.0)	<0.001∗
Skeletal muscle mass (kg), mean ± SD	18.95 ± 3.36^a,b^	18.23 ± 3.28^c,d^	25.40 ± 5.13^a,c^	25.02 ± 5.43^b,d^	<0.001∗
Skeleton muscle mass index (kg/m^2^), mean ± SD	5.72 ± 0.74^a,b^	5.43 ± 0.66^c,d^	7.37 ± 1.02^a,c^	7.02 ± 1.03^b,d^	<0.001∗
M < 7.0 kg/m^2^); F < 5.7 kg/m^2^, n(%)	54 (100.0)	24 (100.0)	6 (3.0)	5 (10.0)	<0.001∗
Body fat mass (kg), median (IQR)	19.75 (17.6, 22.7)^a,b,c^	13.1 (10.3, 17.2)^a,d^	26 (23.5, 32.8)^b,d,e^	16.1 (13.4, 18.8)^c,e^	<0.001∗
Percent body fat mass (%), mean ± SD	36.06 ± 5.80^a,b^	27.47 ± 5.63^a,c^	36.92 ± 6.31^c,d^	25.71 ± 6.53^b,d^	<0.001∗
M ≥ 25%; F ≥ 35%, n(%)	54 (100.0)	0 (0.0)	201 (100.0)	0 (0.0)	<0.001∗
Monofilament, n(%)	14 (25.9)	7 (29.2)	35 (17.4)	9 (18.0)	0.330
Diabetic retinopathy, n(%)	5 (9.3)	5 (20.8)	19 (9.5)	9 (18.0)	0.157
Diabetic nephropathy	33 (61.1)	12 (50.0)	105 (52.2)	21 (42.0)	0.279

a,b,c: Identical letters indicate statistically significant differences between groups at the 0.05 level.

∗Statistically significant at the 0.05 level (α = 0.05).

Laboratory findings showed significant differences among the four groups in fasting plasma glucose, eGFR, higher triglyceride levels, HDL, vitamin D levels, hematocrit, and hemoglobin. The sarcopenic obesity group had significantly lower eGFR, hematocrit, and hemoglobin than the obesity and control groups, as shown in Table S1. Regarding treatment, most patients were prescribed statins (92.3%), metformin (87.5%), RAS inhibitors (58.7%), calcium channel blockers (CCB) (51.9%), and insulin therapy (31.1%). Medication use differed significantly among the four groups for sulfonylureas, calcium, and CCB (P < 0.05). Notably, calcium intake in the sarcopenic obesity group was significantly higher than in the other groups. However, 73.9% of patients were prescribed polypharmacy (Use of more than three antidiabetic drugs in combination with other medications, with a total of more than four drugs.), with the highest proportion observed in the sarcopenia alone group at 79.2%, followed by the obesity group at 77.1%. The differences between the groups were not statistically significant (P = 0.201), as shown in Table S2. Serum hs-CRP levels did not differ significantly across body composition phenotypes (P = 0.63). However, a numerically higher median hs-CRP was observed in participants with obesity alone compared with those with sarcopenic obesity and sarcopenia alone (Table S3).

Univariate logistic regression analysis identified several factors significantly associated with sarcopenic obesity at the 0.05 level, as presented in Table 4. Factors that increased the risk of sarcopenic obesity included older age (Crude OR 1.12 [95% CI 1.05, 1.18]; P < 0.001), no education or only primary schooling (Crude OR 2.63 [95% CI 1.02, 6.75]; P = 0.045), a higher SARC-F score (Crude OR 1.27 [95% CI 1.02, 1.57]; P = 0.032), frailty (Crude OR 5.26 [95% CI 1.42, 19.46]; P = 0.013), smaller calf circumference (Crude OR 1.39 [95% CI 1.18, 1.67]; P < 0.001), smaller neck circumference (Crude OR 1.16 [95% CI 1.02, 1.33]; P = 0.027), longer 4.5-m walking time (Crude OR 1.45 [95% CI 1.12, 1.85]; P = 0.004), longer time for the 5-Time chair stand (Crude OR 1.12 [95% CI 1.03, 1.21]; P = 0.009), higher body fat mass (Crude OR 1.32 [95% CI 1.16, 1.50]; P < 0.001), higher percent body fat (Crude OR 1.30 [95% CI 1.18, 1.43]; P < 0.001), and higher triglyceride levels (Crude OR 1.01 [95% CI 1.00, 1.02]; P = 0.013). Conversely, factors associated with a reduced risk of sarcopenic obesity included longer weekly walking duration (Crude OR 0.94 [95% CI 0.89, 0.99]; P = 0.049), higher systolic blood pressure (Crude OR 0.97 [95% CI 0.57, 0.99]; P = 0.041), elevated diastolic BP (Crude OR 0.95 [95% CI 0.91, 0.99]; P = 0.020), greater hand grip strength (Crude OR 0.90 [95% CI 0.85, 0.95]; P < 0.001), higher skeletal muscle mass (Crude OR 0.73 [95% CI 0.64, 0.83]; P < 0.001), higher skeletal muscle mass index (Crude OR 0.19 [95% CI 0.10, 0.37]; P < 0.001), higher GFR (Crude OR 0.98 [95% CI 0.95, 0.99]; P = 0.035), higher hematocrit levels (Crude OR 0.86 [95% CI 0.77, 0.95]; P = 0.005), and higher hemoglobin levels (Crude OR 0.62 [95% CI 0.45, 0.86]; P = 0.004). No significant associations were found between medication use and sarcopenic obesity (Table 4) (Table S5).

**Table 4:** Univariable analysis of factors associated with sarcopenic obesity.

Factors	Crude OR (95%CI)	P-value
Age (years)	1.12 (1.05, 1.18)	<0.001∗
Sex		
Male	Reference	
Female	1.26 (0.58, 2.73)	0.551
Education		
No education/Primary school	2.63 (1.02, 6.75)	0.045∗
Secondary school or higher	Reference	
Body mass index (BMI)	0.97 (0.83, 1.13)	0.693
Underlying disease, n(%)		
Hypertension	1.23 (0.51, 2.95)	0.648
Dyslipidemia	2.36 (0.86, 6.51)	0.097
Myocardial infarction	1.49 (0.45, 4.93)	0.513
Cancer	1.25 (0.27, 5.90)	0.775
Duration of DM (years)	1.00 (0.97, 1.03)	0.983
Family history of DM	0.63 (0.27, 1.49)	0.296
Smoking	0.44 (0.08, 2.53)	0.359
Alcohol consumption	0.19 (0.05, 0.71)	0.013∗
Herbal use	1.17 (0.30, 4.64)	0.820
Weight loss in 1 year	0.75 (0.34, 1.66)	0.479
Frequency of exercise		
None	1.44 (0.58, 3.58)	0.433
1–2 day/week	1.78 (0.36, 8.76)	0.480
3–4 day/week	0.68 (0.21, 2.21)	0.520
≥5 day/week	Reference	
Walking time (hour/week)	0.94 (0.89, 0.99)	0.049∗
Fatigue	1.46 (0.65, 3.27)	0.356
SARC-F score	1.27 (1.02, 1.57)	0.032∗
Frailty phenotype, n(%)		
Non-frail	Reference	
Pre-frailty	2.24 (0.75, 6.66)	0.147
Frailty	5.26 (1.42, 19.46)	0.013∗
Systolic blood pressure (mmHg)	0.97 (0.57, 0.99)	0.041∗
Elevated diastolic BP (mmHg)	0.95 (0.91, 0.99)	0.020∗
Calf circumference (cm)	1.39 (1.18, 1.67)	<0.001∗
Waist circumference (cm)	1.02 (0.97, 1.07)	0.423
Neck circumference (cm)	1.16 (1.02, 1.33)	0.027∗
4.5-m walking (sec)	1.44 (1.12, 1.85)	0.004∗
5-Time chair stand (sec)	1.12 (1.03, 1.21)	0.009∗
Hand grip strength (kg)	0.90 (0.85, 0.95)	<0.001∗
Skeletal muscle mass (kg)	0.73 (0.64, 0.83)	<0.001∗
Skeleton muscle mass index (kg/m^2^)	0.19 (0.10, 0.37)	<0.001∗
Body fat mass (kg)	1.32 (1.16, 1.50)	<0.001∗
Percent body fat mass (%)	1.30 (1.18, 1.43)	<0.001∗
Monofilament	1.59 (0.62, 4.10)	0.333
Diabetic retinopathy	0.46 (0.14, 1.50)	0.199
Diabetic nephropathy	2.17 (0.99, 4.75)	0.053

The total sample size was 104, with the control group serving as the reference group (n = 91).

∗Statistically significant at the 0.05 level (α = 0.05).

**Fig. 2:**
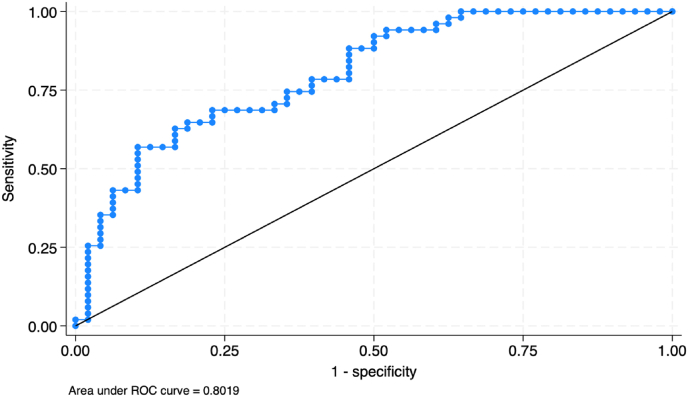
Receiver operating characteristic (ROC) curve analysis for the multivariable model predicting sarcopenic obesity.

Further analysis using multivariable logistic regression with forward stepwise selection identified factors independently associated with sarcopenic obesity. Advancing age remained a significant risk factor, with older patients more likely to develop sarcopenic obesity (Adjusted OR 1.08 [95% CI 1.01, 1.15]; P = 0.017). Conversely, smaller calf circumferences (Adjusted OR 1.35 [95% CI 1.11, 1.67]; P = 0.003) and greater hand grip strength (Adjusted OR 0.93 [95% CI 0.87, 0.99]; P = 0.025) were associated with a reduced risk of sarcopenic obesity. These three predictive factors demonstrated good discrimination in identifying sarcopenic obesity (AUC = 0.802), as shown in Table 5 and Fig. 2.

**Table 5:** Multivariable analysis of factors associated with sarcopenic obesity.

Factors	Adjusted OR (95%CI)	P-value
Age increases 1 year	1.08 (1.01, 1.15)	0.017∗
Calf circumferences decrease 1 cm	1.35 (1.11, 1.67)	0.003∗
Hand grip strength increase 1 kg	0.93 (0.87, 0.99)	0.025∗

Data were analyzed with Multiple logistic regression by forward stepwise method.

The total sample size was 104, with the control group serving as the reference group (n = 50).

∗Statistically significant at the 0.05 level (α = 0.05).

### 3.1 Exploratory analyses of antidiabetic medications

In stratified analyses (Table S4), the prevalence of sarcopenic obesity was lower among GLP-1 receptor agonist users (3/43, 7.0%) than among non-users (49/269, 18.2%), with a borderline association (unadjusted OR 0.34, 95% CI 0.10–1.13; p = 0.066). GLP-1 receptor agonist users also more frequently achieved ≥5% weight loss over 1 year (44.2% vs 29.4%; p = 0.052). In contrast, no significant difference in sarcopenic obesity prevalence was observed according to SGLT2 inhibitor use (14.3% vs 17.8%; p = 0.445).

## 4 Discussion

The objective of this study was to determine the prevalence of sarcopenic obesity (defined as the coexistence of low muscle mass and high body fat) and sarcopenia alone among Thai elderly with T2DM in urban areas, using the AACE 2016 definitions for obesity. Risk factors and associations between sarcopenic obesity, body impedance analysis, and physical performance tests were evaluated. This prevalence was lower than that reported in Zhou’s meta-analysis [17], which estimated an overall prevalence of approximately 26% in Asian populations. However, direct comparisons should be interpreted cautiously because the studies included in the meta-analysis applied heterogeneous definitions of both sarcopenia and obesity (e.g., varying sarcopenia criteria and adiposity thresholds such as BMI- or body fat–based definitions). In contrast, when using comparable diagnostic criteria, our prevalence was similar to that reported by Han et al. [18] in a Chinese cohort. The authors used the AWGS 2019 criteria for sarcopenia and the AACE 2016 criteria for obesity, consistent with the study conducted in China. BMI was higher in the obesity group but normal in the other groups. In cases of sarcopenic obesity, BMI was normal, similar to findings in the previous meta-analysis [17], possibly due to loss of muscle mass and increased body fat mass. Instead of using BMI for screening sarcopenic obesity, body composition analysis should be considered. Sarcopenic obesity was characterized by reduced skeletal muscle mass index, smaller calf and neck circumferences, and poorer physical performance metrics, including slower walking speed and weaker handgrip strength, compared with the other groups.

Frailty was more common in sarcopenic obesity and sarcopenia alone than in other groups. It was observed at 29.2% in sarcopenia alone and 31.5% in sarcopenic obesity, consistent with Sri-on et al. [19]^,^ who studied sarcopenia in people in Thailand. Frailty increased 5.26-fold (crude OR 5.26, 95% CI 1.42–19.46) in sarcopenic obesity, but this was not statistically significant in multivariate analysis. There were no statistically significant differences in walking time, exhaustion, or exercise frequency. HbA1c levels also showed no statistically significant difference, similar to Anna Izzu's review [20]. However, sarcopenic obesity was associated with a higher 5-time chair stand test and lower hand grip strength. Although hs-CRP levels were not statistically different across groups, the observed distribution suggests that low-grade systemic inflammation is present across adverse body composition phenotypes in older adults with T2DM. The lack of a significant difference may reflect limited sample size in the sarcopenia-alone and sarcopenic obesity groups or heterogeneity in inflammatory burden. Nevertheless, chronic inflammation remains a biologically plausible contributor to insulin resistance and muscle catabolism in this population.

Sarcopenic obesity was associated with a significantly lower glomerular filtration rate, consistent with the previous meta-analysis [17]. However, diabetic nephropathy was higher in sarcopenic obesity, but the difference was not statistically significant. In this study, the urine albumin-to-creatinine ratio was similar to that reported by Fuyuko Takahashi in Japan [21]^.^ There was also a lower hemoglobin level compared with the normal group, possibly due to chronic inflammation [1]^.^

From multivariate analysis, advancing age was associated with sarcopenia and sarcopenic obesity, consistent with other studies [20] (aOR 1.08, 95% CI 1.01–1.15). A smaller calf circumference (aOR 1.35, 95% CI 1.11–1.67, P < 0.001) was also associated with sarcopenic obesity, whereas higher handgrip strength (aOR 0.93, 95% CI 0.87–0.99, P < 0.001) was protective. In exploratory analyses, GLP-1 receptor agonist use was associated with a lower prevalence of sarcopenic obesity and a higher frequency of clinically meaningful weight loss. Although not statistically definitive, this pattern is biologically plausible, given the known effects of GLP-1 receptor agonists on appetite regulation, fat mass reduction, and potential muscle preservation. These findings should be interpreted as hypothesis-generating and warrant confirmation in longitudinal studies with detailed body composition assessment.

### 4.1 Strength and limitation section

The strength of this study is that it is the first to examine sarcopenic obesity in people with T2DM, providing prevalence and risk factor data from urban areas of Thailand. However, there are some limitations. First, it excludes patients with neurological impairment, such as stroke, which is a macro complication of T2DM. Second, it includes only patients from urban areas, which may not represent the general population in Thailand. Finally, this cross-sectional study cannot determine the causal relationship between sarcopenic obesity and risk factors.

## 5 Conclusion

Sarcopenic obesity remains difficult to compare across studies due to varying obesity definitions. Based on the AWGS 2019 and adiposity-based criteria, its prevalence in this cohort was 16.4%. Older age and smaller calf circumference were associated with a higher likelihood of sarcopenic obesity, while higher handgrip strength was protective. Interventions emphasizing physical activity, resistance training, and optimized diabetes management should be prioritized. Further studies are needed to determine effective strategies to improve quality of life and reduce morbidity and mortality in people with T2DM and sarcopenic obesity.

- Sarcopenic obesity was prevalent (16.4%) among Thai adults with established T2DM.
- Older age and smaller calf circumference were independent risk factors, while higher handgrip strength was protective.
- Routine screening and interventions emphasizing muscle preservation may help reduce adverse outcomes in this population.

## Declaration of the use of artificial intelligence

No artificial intelligence (AI) tools were used in the preparation of this manuscript.

## Funding

This study was supported by the Thai Osteoporosis Foundation (TOPF) and the Department of Medicine, Phramongkutklao Hospital. The funding did not influence the study design, data collection, analysis, manuscript preparation, or the decision to publish.

## Declaration of competing interest

The authors declare no conflicts of interest.
