# Supplementary material for: Prevalence and risk factors of sarcopenic obesity in Thai people with diabetes: A cross-sectional study
Source: Obes Pillars. 2026 Feb 7;18:100249. doi: 10.1016/j.obpill.2026.100249 (PMC12930160; doi:10.1016/j.obpill.2026.100249)
Supplement: Multimedia component 1 [file mmc1.docx]

**Supplementary materials**

**Table S1** Laboratory of T2DM patients classified by sarcopenia and/or obesity

| **Variables** | **Sarcopenic obesity** | **Sarcopenia alone** | **Obesity** | **Control** | **P-value** |
| --- | --- | --- | --- | --- | --- |
| **n (%) [95%CI]** | **54 (16.4%)**  **[12.8%, 20.8%]** | **24 (7.3%)**  **[4.9%, 10.7%]** | **201 (61.1%)**  **[55.7%, 66.2%]** | **50 (15.2%)**  **[11.7%, 19.5%]** |  |
| Fasting plasma glucose (mg/dL), median (IQR) | 118 (102, 134)  n=51 | 110 (95, 139)  n=23 | 122 (104, 147)^a^  n=191 | 110 (92, 128)^a^  n=46 | 0.004* |
| HbA1C (%), median (IQR) | 7.0 (6.1, 7.7)  n=51 | 7.0 (6.6, 7.6)  n=23 | 6.9 (6.4, 7.7)  n=191 | 7.0 (6.1, 7.6)  n=46 | 0.807 |
| BUN (mg/dL), median (IQR) | 17.1 (13.7, 20.4)  n=51 | 18.5 (12.8, 26.2)  n=23 | 15.3 (12.5, 20.2)  n=191 | 16.8 (13.7, 19.9)  n=46 | 0.358 |
| Cr (mg/dL), median (IQR) | 1.02 (0.88, 1.22)  n=51 | 0.91 (0.68, 1.26)  n=23 | 0.98 (0.79, 1.22)  n=191 | 0.93 (0.82, 1.11)  n=46 | 0.579 |
| GFR (mL/min/1.73 m²), median (IQR) | 59 (50.6, 76.9)^a,b^  n=51 | 65.7 (47.5, 84.7)  n=26 | 71.3 (56.2, 86.0)^a^  n=191 | 70.52 (58.6, 86.3)^b^  n=46 | 0.020* |
| Serum albumin (g/dL), median (IQR) | 4.26 (4.0, 4.43)  n=51 | 4.34 (4.12, 4.49)  n=22 | 4.30 (4.1, 4.44)  n=180 | 4.25 (4.09, 4.46)  n=43 | 0.885 |
| Total bilirubin (mg/dL), median (IQR) | 0.49 (0.37, 0.62)  n=50 | 0.39 (0.33, 0.61)  n=21 | 0.52 (0.40, 0.71)  n=177 | 0.43 (0.39, 0.63)  n=43 | 0.079 |
| Direct bilirubin (mg/dL), median (IQR) | 0.23 (0.17, 0.28)  n=50 | 0.21 (0.15, 0.27)  n=21 | 0.24 (0.19, 0.32)  n=177 | 0.21 (0.15, 0.29)  n=43 | 0.093 |
| AST (U/L), median (IQR) | 24 (20, 31)  n=51 | 25 (20, 32)  n=23 | 24 (21. 29)  n=187 | 26 (20, 32.5)  n=44 | 0.827 |
| ALT (U/L), median (IQR) | 18 (13, 25)  n=51 | 18.4 (11, 28)  n=23 | 21 (14, 28)  n=187 | 22 (16, 35)  n=45 | 0.218 |
| ALP (U/L), median (IQR) | 71 (57, 95)  n=50 | 78 (70, 98)  n=22 | 76 (62, 93)  n=178 | 67 (55, 87)  n=44 | 0.138 |
| Uric acid (mg/dL), median (IQR) | 5.8 (4.5, 7.2)  n=37 | 4.6 (4.1, 5.4)  n=13 | 5.7 (4.7, 6.6)  n=131 | 5.7 (4.9, 6.4)  n=29 | 0.239 |
| Total cholesterol (mg/dL), median (IQR) | 154 (135, 177)  n=52 | 162 (144, 188)  n=22 | 150 (132, 172)  n=189 | 152 (135, 169)  n=45 | 0.216 |
| Triglyceride (mg/dL), median (IQR) | 117 (93, 174)^a,b^  n=52 | 89 (69, 138)^a,c^  n=22 | 115 (86, 149)^c,d^  n=189 | 89 (77, 115)^b,d^  n=45 | 0.002* |
| HDL (mg/dL), median (IQR) | 55 (47, 65)  n=52 | 60 (53, 64)^a^  n=22 | 52 (45, 64)^a,b^  n=189 | 59 (50, 66)^b^  n=45 | 0.032* |
| LDL (mg/dL), median (IQR) | 80 (69, 103)  n=52 | 88 (78, 106)  n=22 | 83 (65, 99)  n=189 | 83 (66, 103)  n=46 | 0.725 |
| Vitamin D level (ng/dL), median (IQR) | 38 (28.7, 48.3)  n=33 | 44.2 (32.5, 67.1)^a,b^  n=18 | 35.1 (27.5, 44)^a^  n=142 | 37.7 (24.7, 49.7)^b^  n=33 | 0.039* |
| UACR (mg/g) | 14.84 (6.62, 80.42)  n=49 | 22.27 (8.53, 66.44)  n=22 | 17.59 (6.57, 57.6)  n=187 | 10.89 (4.32, 37.90)  n=41 | 0.245 |
| Hematocrit (%), mean±SD | 37.41±4.95^a,b^  n=50 | 37.37±4.65^c^  n=23 | 40.28±4.39^a,c^  n=179 | 40.23±3.83^b^  n=45 | <0.001* |
| Hemoglobin (g/dL), mean±SD | 12.23±1.47^a,b^  n=50 | 12.23±1.64^c^  n=23 | 13.18±1.50^a,c^  n=179 | 13.14±1.34^b^  n=45 | <0.001* |

a,b,c: Identical letters indicate statistically significant differences between groups at the 0.05 level.

*Statistically significant at the 0.05 level (α=0.05).

**Table S2** Medication of T2DM patient classified by sarcopenia and/or obesity

| **Variables** | **Sarcopenic obesity** | **Sarcopenia alone** | **Obesity** | **Control** | **P-value** |
| --- | --- | --- | --- | --- | --- |
| **n (%) [95%CI]** | **54 (16.4%)**  **[12.8%, 20.8%]** | **24 (7.3%)**  **[4.9%, 10.7%]** | **201 (61.1%)**  **[55.7%, 66.2%]** | **50 (15.2%)**  **[11.7%, 19.5%]** |  |
| Metformin | 44 (84.6) | 19 (82.6) | 167 (87.4) | 43 (93.5) | 0.493 |
| Sulfonylurea | 14 (26.9) | 9 (39.1) | 80 (41.9) | 9 (19.6) | 0.017* |
| SGLT-2i | 14 (26.9) | 6 (26.1) | 62 (32.5) | 16 (34.8) | 0.770 |
| GLP1-RA | 3 (5.8) | 3 (13.0) | 33 (17.3) | 4 (8.7) | 0.123 |
| DPP-4i | 22 (42.3) | 8 (34.8) | 49 (25.7) | 13 (28.3) | 0.122 |
| Pioglitazone | 7 (13.5) | 2 (8.7) | 36 (18.9) | 5 (10.9) | 0.360 |
| Insulin | 17 (32.7) | 9 (39.1) | 57 (29.8) | 14 (30.4) | 0.824 |
| Statin | 49 (94.2) | 19 (82.6) | 179 (93.7) | 41 (89.1) | 0.212 |
| Antiplatelet | 23 (44.1) | 11 (47.8) | 73 (38.2) | 18 (39.1) | 0.743 |
| Bisphosphonate | 0 (0.0) | 2 (8.7) | 5 (2.6) | 2 (4.4) | 0.194 |
| Denosumab | 4 (1.3) | 0 (0.0) | 2 (1.1) | 0 (0.0) | 0.298 |
| Vitamin D | 22 (42.3) | 12 (52.2) | 90 (52.2) | 20 (43.5) | 0.838 |
| Steroid | 2 (3.9) | 0 (0.0) | 3 (1.6) | 0 (0.0) | 0.426 |
| Calcium | 19 (36.5) | 7 (30.4) | 30 (15.7) | 9 (19.6) | 0.007* |
| RAS inhibitor | 27 (51.9) | 10 (43.5) | 120 (62.8) | 26 (56.5) | 0.202 |
| CCB | 21 (40.4) | 11 (47.8) | 112 (58.6) | 18 (39.1) | 0.024* |
| Diuretic | 7 (13.5) | 0 (0.0) | 20 (10.5) | 3 (6.5) | 0.262 |
| Beta blocker | 8 (15.4) | 1 (4.4) | 42 (22.0) | 7 (15.2) | 0.155 |
| Alpha blocker | 0 (0.0) | 1 (4.4) | 13 (6.8) | 1 (2.2) | 0.172 |
| Polypharmacy | 35 (64.8) | 19 (79.2) | 155 (77.1) | 34 (68.0) | 0.201 |
|  |  |  |  |  |  |

n=312; Data were expressed as n (%).

*Statistically significant at the 0.05 level (α=0.05).

**Table S3 High-sensitivity C-reactive protein levels according to sarcopenia and obesity phenotypes**

| Group | n | hs-CRP, median (IQR) mg/L |
| --- | --- | --- |
| Sarcopenic obesity | 9 | 0.58 (0.42–1.53) |
| Obesity alone | 34 | 1.12 (0.42–3.09) |
| Sarcopenia alone | 4 | 0.66 (0.40–1.11) |

High-sensitivity C-reactive protein (hs-CRP) levels are presented as median and interquartile range (IQR) according to sarcopenia and obesity phenotypes. Sarcopenia was defined using the Asian Working Group for Sarcopenia (AWGS) 2019 criteria, and obesity was defined by body fat percentage. The analysis explores the inflammatory–muscle axis in older adults with type 2 diabetes and is intended to be hypothesis-generating. Due to the small sample size in some subgroups, formal statistical comparisons were not emphasized.

**Table S4 Sarcopenic obesity and ≥5% weight loss according to modern antidiabetic therapy**

| Medication | Users (n) | SO n (%) | Non-users (n) | SO n (%) | Unadjusted OR (95% CI) | p |
| --- | --- | --- | --- | --- | --- | --- |
| SGLT2 inhibitor | 98 | 14 (14.3) | 214 | 38 (17.8) | 0.77 (0.40–1.50) | 0.445 |
| GLP-1 receptor agonist | 43 | 3 (7.0) | 269 | 49 (18.2) | 0.34 (0.10–1.13) | 0.066 |
| **Secondary outcome: ≥5% weight loss in 1 year** | | | | | | |
| Medication | Users: ≥5% WL (%) | Non-users: ≥5% WL (%) | Unadjusted OR | p |  |  |
| SGLT2 inhibitor | 37.8 | 28.5 | 1.52 | 0.102 |  |  |
| GLP-1 receptor agonist | 44.2 | 29.4 | 1.90 | 0.052 |  |  |

Sarcopenic obesity (SO) was defined as sarcopenia (AWGS 2019; ASW cut-off) plus obesity defined by body fat percentage. Analyses are hypothesis-generating due to cross-sectional design and potential confounding by indication.

**Table S5** Univariable analysis of laboratory and medication factors associated with sarcopenic obesity

| **Factors** | **Crude OR (95%CI)** | **P-value** |
| --- | --- | --- |
| Fasting plasma glucose (mg/dL) *(n=97)* | 1.01 (0.99, 1.02) | 0.254 |
| HbA1C (%) *(n=97)* | 1.13 (0.85, 1.50) | 0.407 |
| BUN (mg/dL) *(n=97)* | 1.00 (0.96, 1.05) | 0.930 |
| Cr (mg/dL) *(n=97)* | 1.72 (0.59, 5.04) | 0.323 |
| GFR (mL/min/1.73 m²) *(n=97)* | 0.98 (0.95, 0.99) | 0.035* |
| Serum albumin (g/dL) *(n=94)* | 0.53 (0.14, 2.08) | 0.364 |
| Total bilirubin (mg/dL) *(n=93)* | 0.67 (0.13, 3.45) | 0.630 |
| Direct bilirubin (mg/dL) *(n=93)* | 0.53 (0.01, 19.98) | 0.731 |
| AST (U/L) *(n=95)* | 0.98 (0.95, 1.02) | 0.322 |
| ALT (U/L) *(n=96)* | 0.98 (0.96, 1.01) | 0.169 |
| ALP (U/L) *(n=109)* | 1.01 (0.99, 1.02) | 0.211 |
| Uric acid (mg/dL) *(n=66)* | 1.08 (0.77, 1.52) | 0.643 |
| Total cholesterol (mg/dL) *(n=97)* | 1.00 (0.99, 1.01) | 0.775 |
| Triglyceride (mg/dL) *(n=97)* | 1.01 (1.00, 1.02) | 0.013* |
| HDL (mg/dL) *(n=97)* | 0.99 (0.96, 1.02) | 0.524 |
| LDL (mg/dL) *(n=98)* | 1.00 (0.99, 1.01) | 0.999 |
| Vitamin D level (ng/dL) *(n=66)* | 1.01 (0.98, 1.04) | 0.704 |
| UACR (mg/g) *(n=90)* | 1.00 (0.99, 1.00) | 0.682 |
| Hematocrit (%) *(n=95)* | 0.86 (0.77, 0.95) | 0.005* |
| Hemoglobin (g/dL) *(n=95)* | 0.62 (0.45, 0.86) | 0.004* |
| Metformin | 0.38 (0.10, 1.54) | 0.177 |
| Sulfonylurea | 1.51 (0.58, 3.92) | 0.393 |
| SGLT-2i | 0.69 (0.29, 1.64) | 0.400 |
| GLP1 RA | 0.64 (0.14, 3.04) | 0.577 |
| DPP-4i | 1.86 (0.80, 4.36) | 0.150 |
| Pioglitazone | 1.28 (0.38, 4.33) | 0.687 |
| Insulin | 1.11 (0.47, 2.61) | 0.810 |
| Statin | 1.99 (0.45, 8.84) | 0.365 |
| RAS inhibitor | 0.83 (0.37, 1.84) | 0.649 |
| Antiplatelet | 1.23 (0.55, 2.76) | 0.610 |
| Vitamin D | 0.95 (0.43, 2.12) | 0.907 |
| Calcium | 2.37 (0.94, 5.95) | 0.067 |
| CCB | 1.05 (0.47, 2.37) | 0.899 |
| Diuretic | 2.23 (0.54, 9.18) | 0.267 |
| Beta blocker | 1.01 (0.34, 3.05) | 0.982 |
| Polypharmacy | 0.87 (0.38, 1.96) | 0.731 |

The total sample size was 104, with the control group serving as the reference group (n=91).

*Statistically significant at the 0.05 level (α=0.05).
